# Supplementary material for: Nitrogen-Rich Triazine-Based Covalent Organic Frameworks as Efficient Visible Light Photocatalysts for Hydrogen Peroxide Production
Source: Nanomaterials (Basel). 2024 Apr 8;14(7):643. doi: 10.3390/nano14070643 (PMC11013763; doi:10.3390/nano14070643)
Supplement: Supplementary file 1 [file nanomaterials-14-00643-s001.zip › nanomaterials-2934755-supplementary.pdf]

Supplementay Materials

Shu Yang[1,2,a], Keke Zhi[3,4,a], Zhimin Zhang[1,2], Rukiya·Kerem[1,2], Qiong Hong[1,2], Lei Zhao[1,2], Wenbo Wu[1], Lulu Wang1,2,*, and Duozhi Wang1,2,*

1 College of Chemistry, Xinjiang University, Urumqi, 830017, PR China
2 State Key Laboratory of Chemistry and Utilization of Carbon Based Energy Resources,Urumqi, 830017, PR China
3 China University of Petroleum-Beijing at Karamay, Karamay 834000, PR China
4 State Key Laboratory of Heavy Oil Processing-Karamay Branch, Karamay 834000, PR China
* Correspondence: 316813906@qq.com; Tel.:13579275610; xjwangdz@sina.com
a These authors contribute equally to this work.

Materials and Reagents:

1,4-dioxane (AR, > 99%) and methanol were sourced from Tianjin Zhiyuan Chemical Reagent Co., Ltd.(Tientsin China), 1-Butanol (AR, > 99%) and 1,2-Dichlorobenzene (AR, > 98%) were sourced from Greagent(Shanghai China)，5-Bromopicolinonitrile (AR, > 98%) and 4-Bromobenzonitrile(AR, > 98%) were purchased from Adamas Reagent Co., Ltd. (Shanghai, China)，Mesitylene (AR, > 97%) was purchased from Adamas Reagent Co., Ltd. (Shanghai, China), 4-(4,4,5,5-Tetramethyl-1,3,2-dioxaborolan-2-yl)aniline (AR,>98%) was purchased from Adamas Reagent Co., Ltd. (Shanghai, China)，Tetrahydrofuran was sourced from Tianjin Zhiyuan Chemical Reagent Co., Ltd.(Tientsin，China), Tetrakis(triphenylphosphine)palladium (AR, > 99%) was purchased from Adamas Reagent Co., Ltd. (Shanghai, China), 4-Formylphenylboronic Acid (AR, > 97%)，N,N-dimethylformamide (AR, > 99%) was purchased from Adamas Reagent Co., Ltd. (Shanghai, China), dimethyl-1-pyrroline N-oxide (DMPO) and Cesium Fluoride (AR, > 99%) was purchased from Aladdin Co. Ltd., (Shanghai, China). All chemicals were used without further purification.

Experimental Section

Calculation of average fluorescence lifetime

To shed more light on this issue, the TRPL decay plots were fitted through the biexponential decay function presented as follows：

$$R(t) = B_1 \exp\left(-\frac{t}{\tau 1}\right) + B_2 \exp\left(-\frac{t}{\tau 2}\right)$$

$$\tau Ave = (B_1\tau 1^2 + B_2\tau 2^2)/(B_1\tau 1 + B_2\tau 2)$$

where B1 and B2 are the weighing factors, and τ1 and τ2 are the corresponding lifetimes, respectively.

H_2O_2 detection methods.

The amount of H_2O_2 was determined using iodometry. A solution was prepared by adding 0.5 mL of 0.4 mol L^{-1} potassium hydrogen phthalate ($C_8H_5KO_4$) aqueous solution and 0.5 mL of 0.4 mol L^{-1} potassium iodide (KI) aqueous solution. The solution was then kept for 30 min. The H_2O_2 molecules reacted with iodide anions (I^-) under acidic

conditions ($H_2O_2 + 3I^- + 2H^+ \rightarrow I^{3-} + 2H_2O$) to produce triiodide anions. The amount of H_2O_2 was determined by measuring the absorbance at 352 nm using of UV-vis spectroscopy.The linear calibration curve for H_2O_2 concentration was generated by diluting a 9.12 mol L^{-1} H_2O_2 stock solution to cover the range of H_2O_2 concentrations (0-100 μM). The linear relationship between H_2O_2 concentration and the absorption intensity was established as follows:

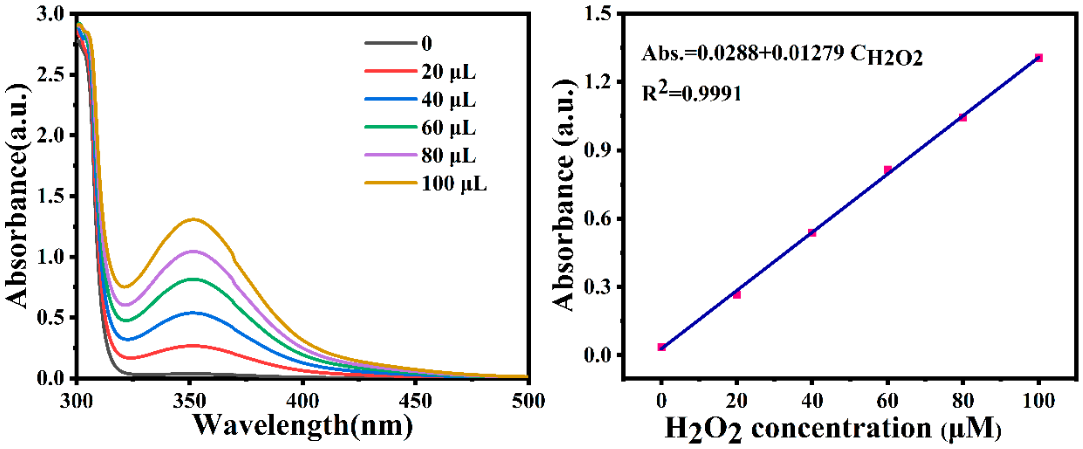

Photocatalytic decomposition of H_2O_2 by COFs.

The photocatalytic consumption of H_2O_2 was tested by dispersing COFs-based catalysts (2.5 mg) in an aqueous solution (10 mL) containing H_2O_2 (50 μM) under continuous N_2 aeration.

Cycling Tests.

The tube was filled with 2.5 mg of photocatalyst, 10 mL of H_2O, and sealed with a septum. The resulting suspension was treated with ultrasonication until the photocatalyst was dispersed, and then degassed by bubbling O_2 for 15 minutes. Next, the mixture solution was stirred under dark conditions for 15 min to achieve the absorption-desorption equilibrium. The reaction mixture was illuminated with 10 W 420 nm LED for 2 h. All the reaction system was kept at 25 °C as controlled by cooling water. After the reaction, the COFs-based photocatalyst was centrifuged, washed with methanol, and dried in a vacuum oven at 70 °C for 12 h. The resulting photocatalyst was then used directly for the subsequent runs.

Electrochemical measurements

Electrochemical measurements of the photocatalysts were performed on a standard three-electrode system by an electrochemical workstation (CHI-660D, Shanghai Chenhua, China). The resulting electrode, twisted platinum wire and Ag/AgCl electrode were used as working, counter and reference electrodes, respectively. The 0.5 M Na_2SO_4 aqueous solution (pH = 7) was chosen as supporting electrolyte.

Preparation of COF film for electrochemical tests: 5 mg of COF sample was dissolved in a mixed solution of 655 μL ethanol, 325 μL H_2O and 20 μL Nafion, and then sonicated for 3 h to form the homogeneous suspension. The obtained suspension was spin-coated on FTO glass, the effective working area of 1 cm^2.

Electron paramagnetic resonance (EPR) measurements

Hydroxyl radical detection: 5,5-dimethyl-1-pyrroline-N-oxide (DMPO) was selected as the trapping agent during the measurements. 50 μL of the H_2O with the 1 mg COF sample was mixed with 5 μL of DMPO trapping agent solution (10 mM). After being irradiated for 300 s with 10 W 420 nm LED visible light, the mixed solution was characterized by an electron spin resonance spectrometer. Superoxide radical detection: 50μL of the MeOH with the 1 mg COF sample was mixed with 5 μL of DMPO trapping agent solution (10 mM). After being irradiated for 300 s with visible light, then the mixed solution was detected.

Supplemental Results

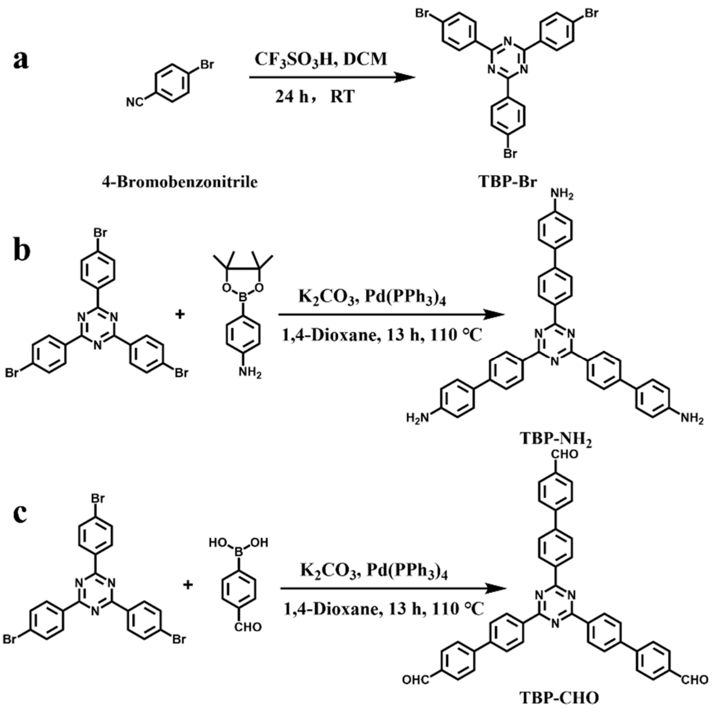

Figure S1. Synthesis routes of: (**a**) TBP-Br; (**b**) TBP-NH_2; (**c**) TBP-CHO.

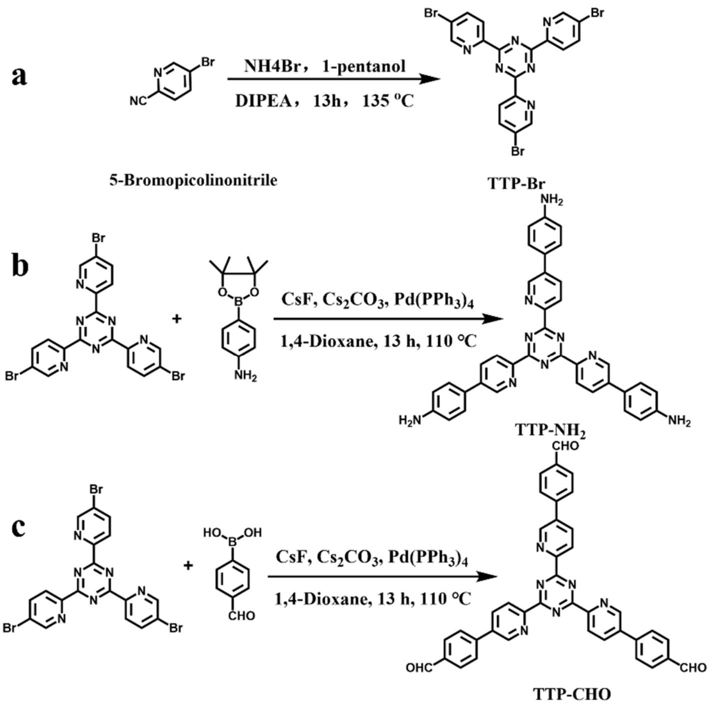

Figure S2. Synthesis routes of: (**a**) TTP-Br; (**b**) TTP-NH2; (**c**) TTP-CHO.

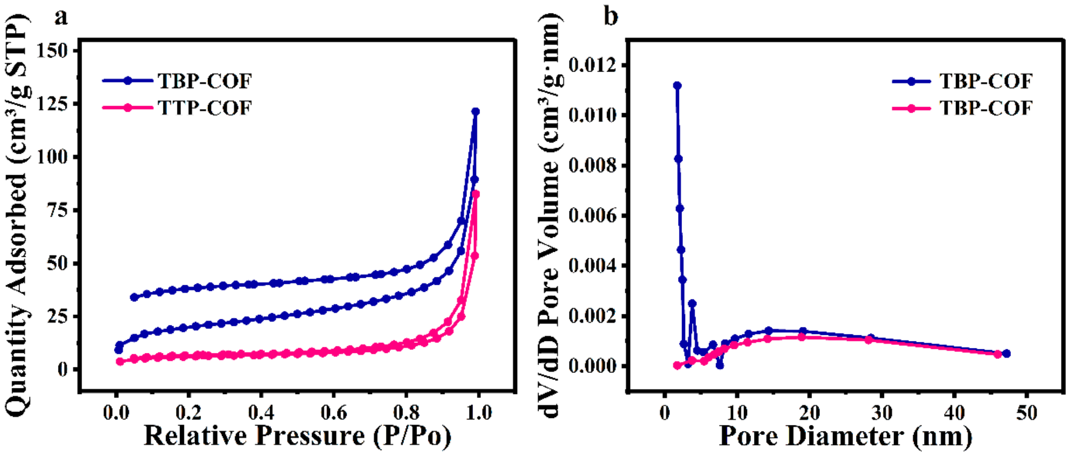

Figure S3. TBP-COF and TTP-COF: (a) N_2 sorption isotherms at 77 K; (b) pore size distributions

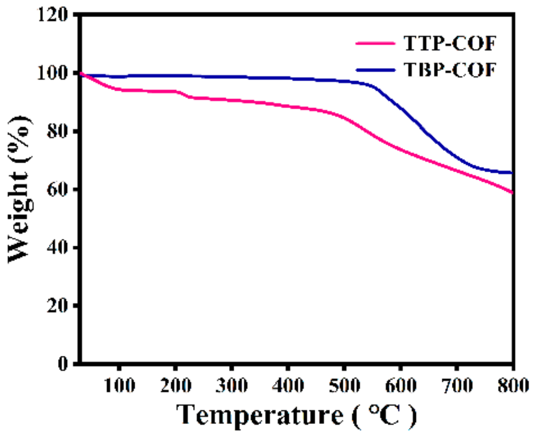

Figure S4. TG curves of TBP-COF and TTP-COF under N_2 atmosphere.

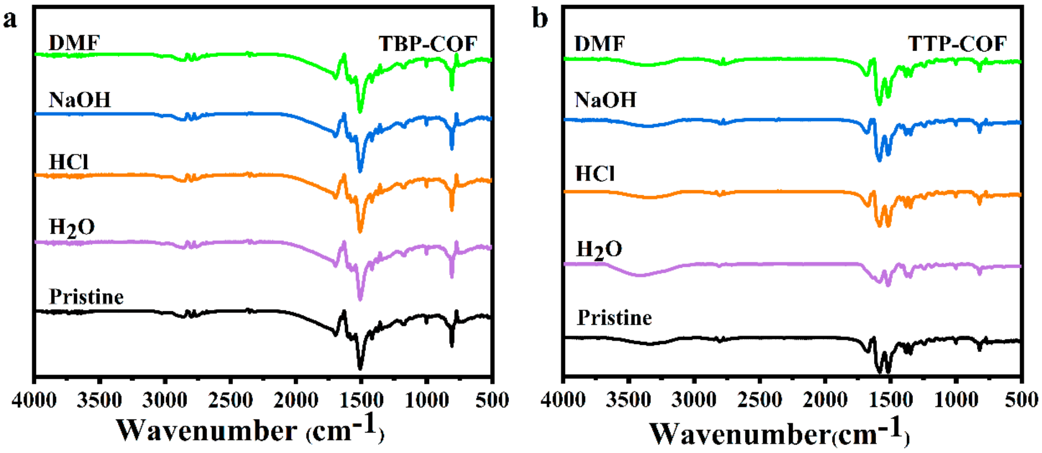

Figure S5. FT-IR patterns of (a) TBP-COF and (b) TTP-COF samples treated at room temperature for 24 h in 1 M of HCl, 1 M of NaOH, H_2O and DMF. The chemical stability of the COFs was evaluated by soaking the COFs in various solvents such as HCl, NaOH, H_2O and DMF at concentrations of 0.5 g L^{-1}.

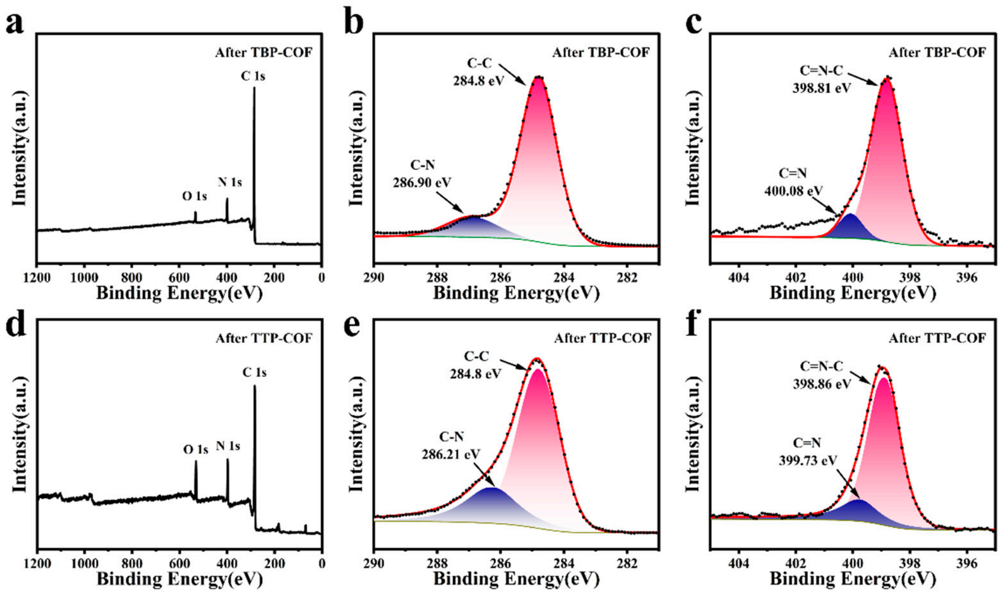

Figure S6. XPS spectra of TBP-COF after photocatalysis: (a) Survey scan; (b) C 1s; (c) N 1s; XPS spectra of TTP-COF after photocatalysis: (d) Survey scan; (e) C 1s; (f) N 1s.

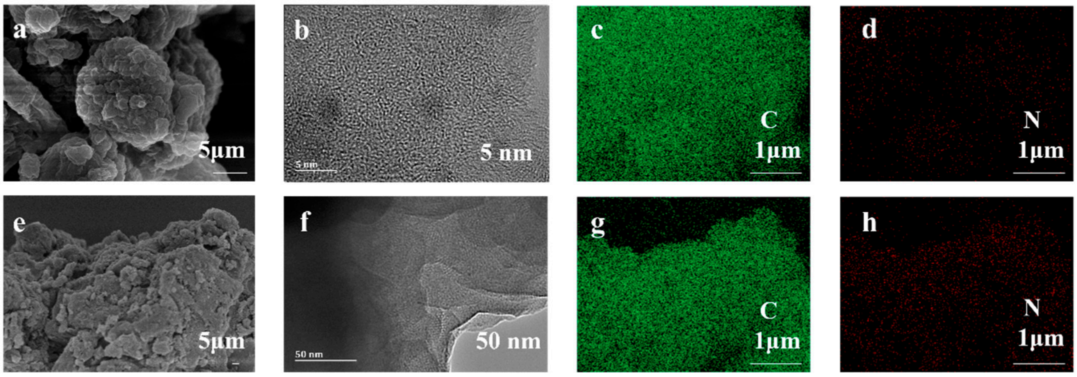

Figure S7. TBP-COF after photocatalysis :(a) SEM; (b) TEM; (c and d) EDS; TTP-COF after photocatalysis :(a) SEM; (b) TEM; (c and d) EDS

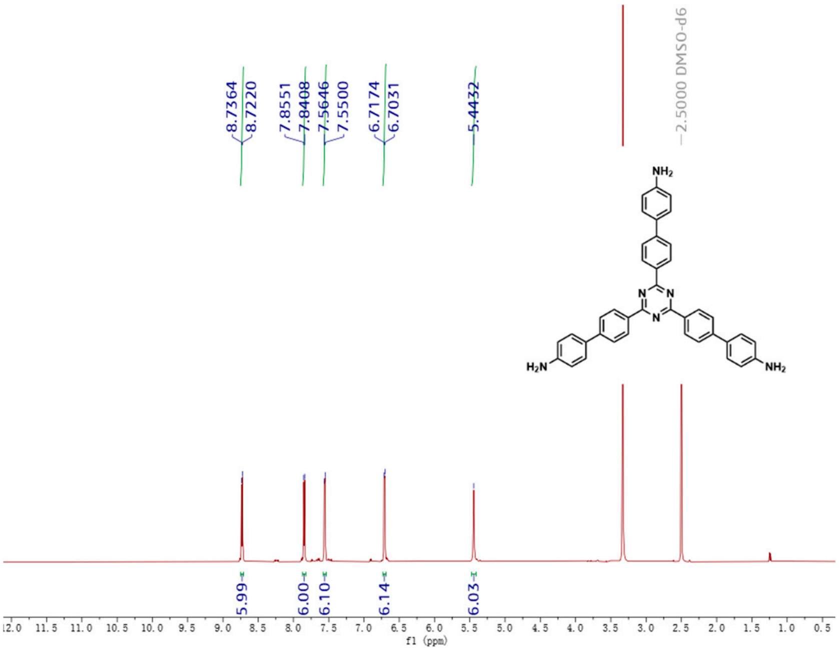

Figure S8. ^1H NMR spectra of TBP-NH_2

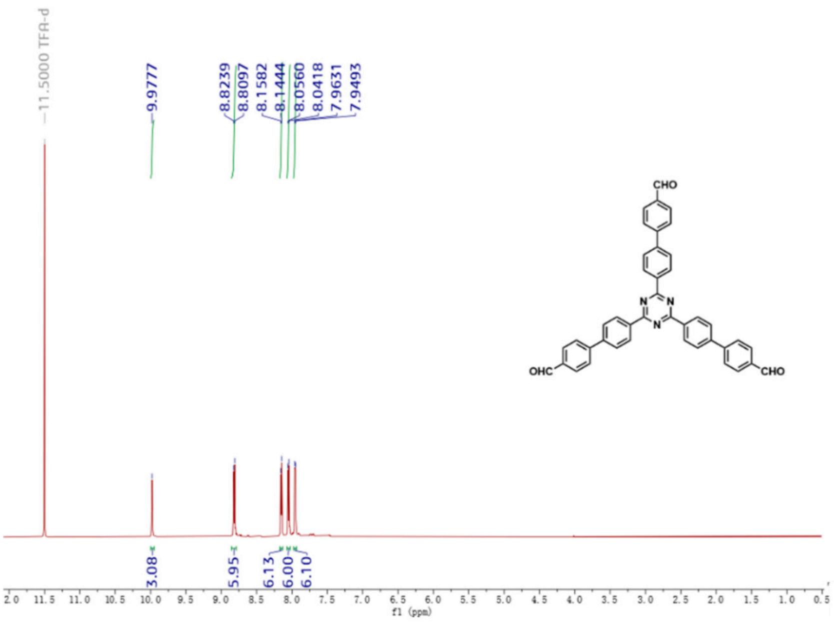

Figure S9. ^{1}H NMR spectra of TBP-CHO

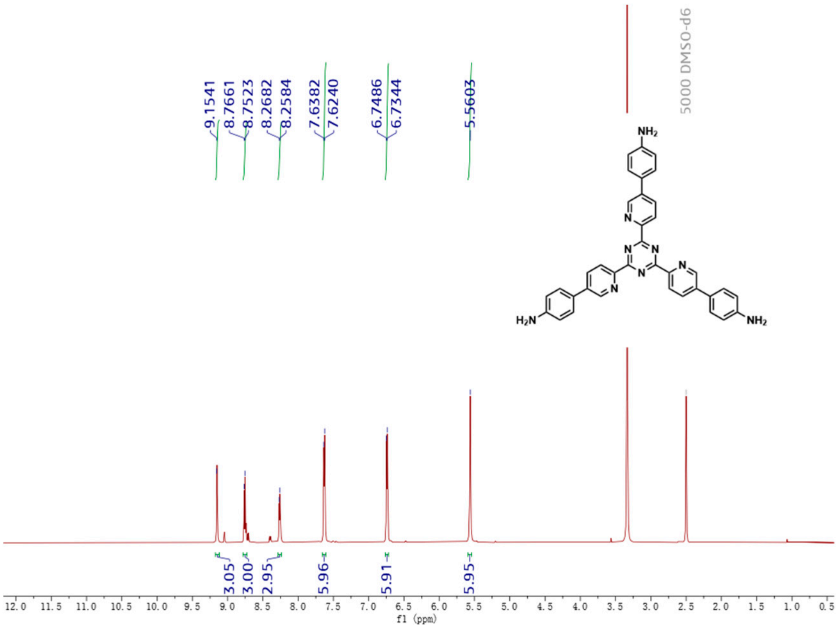

Figure S10. ^{1}H NMR spectra of TTP-NH_2

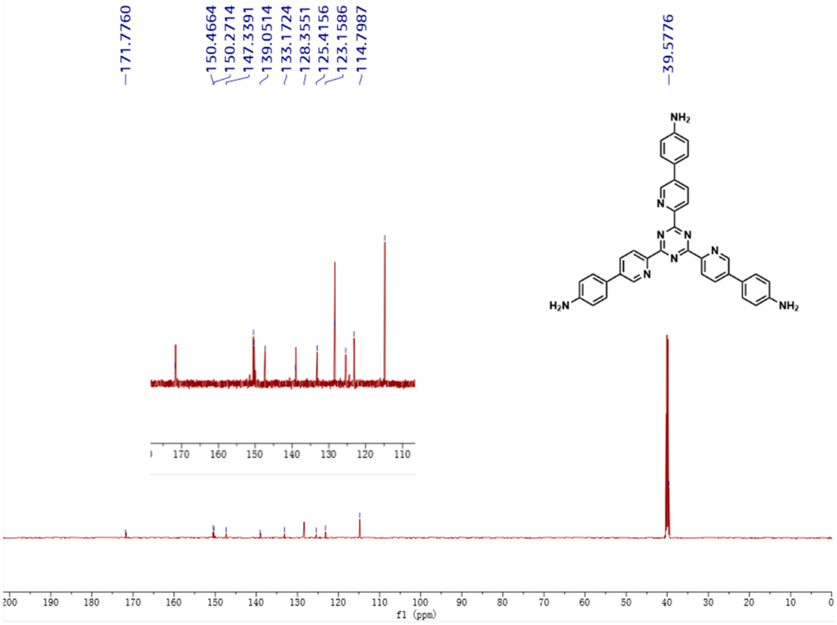

Figure S11. ^{13}C NMR spectra of TTP-NH_2

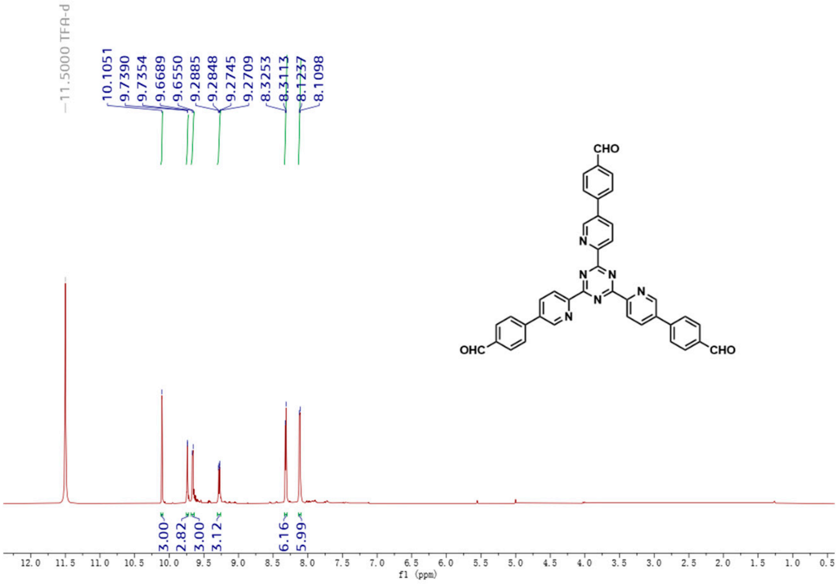

Figure S12. ^{1}H NMR spectra of TTP-CHO

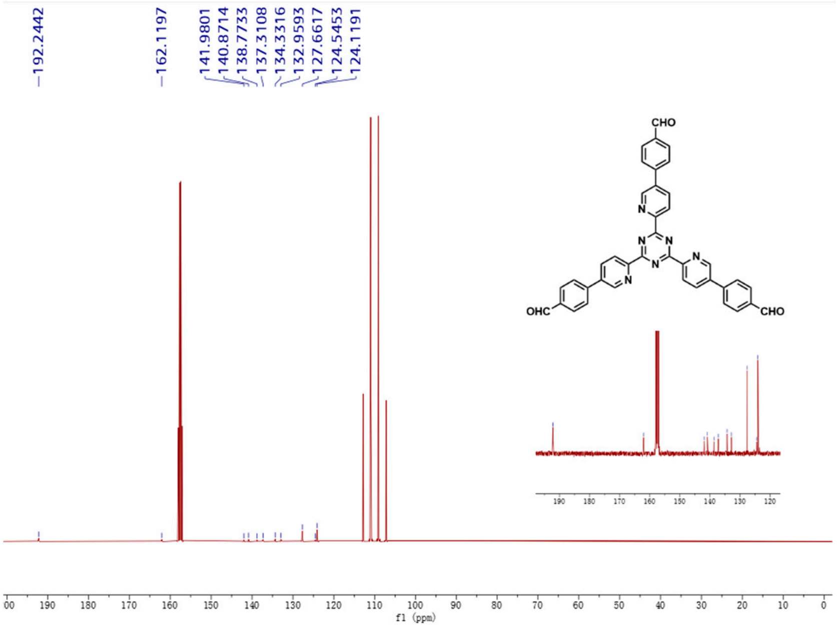

Figure S13. ^{13}C NMR spectra of TTP-CHO

Table S1. Comparison of photocatalytic H_2O_2 performance on various representative photocatalysts.

Photocatalyst	Solvent condition	Photocatalytic activity (μmol g^{-1} h^{-1})	Irradiated conditions	Reference
**TBP-COF**	**H_2O**	**4244**	**LED λ=420nm**	**This work**
**TDB-COF**	**H_2O**	**1868**	**LED λ=420nm**	**This work**
COF-TfpBpy	H_2O	1042	λ > 420 nm	[1]
SonoCOF-F2	H_2O	1244	1-sun λ>420nm	[2]
TPB-DMTP-COF	H_2O	1565	300W Xe λ>420nm	[3]
TF_{50}-COF	H_2O: EtOH (9:1)	1739	> 400 nm	[4]
EBA-COF	H_2O: EtOH (9:1)	1830	LED λ=420nm	[5]
CTF-BPDCN	H_2O	1944	λ>420nm	[6]
COF TTA-TTTA	H_2O	2406	50W LED λ=420nm	[7]
AQTEE-COP	H_2O	3204	300W Xe λ>420nm	[8]
FS-COF	H_2O	3904	300W Xe λ>420nm	[9]
iCOF-DBTP	H_2O	10010	1-sun λ>420nm	[10]

1. Kou, M.; Wang, Y.; Xu, Y.; Ye, L.; Hu10.ang, Y.; Jia, B.; Li, H.; Ren, J.; Deng, Y.; Chen, J. *et al*: Molecularly Engineered Covalent Organic Frameworks for Hydrogen Peroxide Photosynthesis. *Angew Chem Int Ed Engl* **2022**, 61(19):e202200413.[DOI: 10.1002/anie.202200413]
2. Zhao, W.; Yan, P.; Li, B.; Bahri, M.; Liu, L.; Zhou, X.; Clowes, R.; Browning, N.D.; Wu, Y.; Ward, J.W. *et al*: Accelerated Synthesis and Discovery of Covalent Organic Framework Photocatalysts for Hydrogen Peroxide Production. *J Am Chem Soc* **2022**, 144(22):9902-9909. [DOI: 10.1021/jacs.2c02666]
3. Li, L.; Xu, L.; Hu, Z.; Yu, J.C.: Enhanced Mass Transfer of Oxygen through a Gas–Liquid–Solid Interface for Photocatalytic Hydrogen Peroxide Production. *Adv. Funct. Mater* **2021**, 31(52). [DOI: 10.1002/adfm.202106120]
4. Wang, H.; Yang, C.; Chen, F.; Zheng, G.; Han, Q.: A Crystalline Partially Fluorinated Triazine Covalent Organic Framework for Efficient Photosynthesis of Hydrogen Peroxide. *Angew Chem Int Ed Engl* **2022**, 61(19):e202202328. [DOI: 10.1002/anie.202202328]
5. Zhai, L.; Xie, Z.; Cui, C.-X.; Yang, X.; Xu, Q.; Ke, X.; Liu, M.; Qu, L.-B.; Chen, X.; Mi, L.: Constructing Synergistic Triazine and Acetylene Cores in Fully Conjugated Covalent Organic Frameworks for Cascade Photocatalytic H_2O_2 Production. *Chem. Mater.* **2022**, 34(11):5232-5240. [DOI: 10.1021/acs.chemmater.2c00910]
6. Chen, L.; Wang, L.; Wan, Y.; Zhang, Y.; Qi, Z.; Wu, X.; Xu, H.: Acetylene and Diacetylene Functionalized Covalent Triazine Frameworks as Metal-Free Photocatalysts for Hydrogen Peroxide Production: A New Two-Electron Water Oxidation Pathway. *Adv Mater* **2020**, 32(2):e1904433. [DOI: 10.1002/adma.201904433]
7. Tan, F.; Zheng, Y.; Zhou, Z.; Wang, H.; Dong, X.; Yang, J.; Ou, Z.; Qi, H.; Liu, W.; Zheng, Z. *et al*: Aqueous Synthesis of Covalent Organic Frameworks as Photocatalysts for Hydrogen Peroxide Production. *CCS Chem.* **2022**, 4(12):3751-3761. [DOI: 10.31635/ccschem.022.202101578]
8. Xu, X.; Sa, R.; Huang, W.; Sui, Y.; Chen, W.; Zhou, G.; Li, X.; Li, Y.; Zhong, H.: Conjugated Organic Polymers with Anthraquinone Redox Centers for Efficient Photocatalytic Hydrogen Peroxide Production from Water and Oxygen under Visible Light Irradiation without Any Additives. *ACS Catal.* **2022**, 12(20):12954-12963. [DOI: 10.1021/acscatal.2c04085]
9. Luo, Y.; Zhang, B.; Liu, C.; Xia, D.; Ou, X.; Cai, Y.; Zhou, Y.; Jiang, J.; Han, B.: Sulfone-Modified Covalent Organic Frameworks Enabling Efficient Photocatalytic Hydrogen Peroxide Generation Via One-Step Two-Electron O_2 Reduction. *Angew Chem Int Ed Engl* **2023**, 62(26):e202305355. [DOI: 10.1002/anie.202305355]
10. Li, G.; Fu, P.; Yue, Q.; Ma, F.; Zhao, X.; Dong, S.; Han, X.; Zhou, Y.; Wang, J.: Boosting Exciton Dissociation by Regulating Dielectric Constant in Covalent Organic Framework for Photocatalysis. *Chem Catalysis* **2022**, 2(7):1734-1747. [DOI: 10.1016/j.checat.2022.05.002]
